# Baroreflex Sensitivity and Hemodynamic Parameters at Rest and in Response to Physical and Mental Stress: A Comparative Study of Patients Undergoing Different Dialysis Modalities

**DOI:** 10.7759/cureus.82804

**Published:** 2025-04-22

**Authors:** Marieta Theodorakopoulou, Artemios G Karagiannidis, Danai Faitatzidou, Konstantina Dipla, Aggelos Koutlas, Fotini Iatridi, Chrysostomos Dimitriadis, Ioannis Tsouchnikas, Andreas Zafeiridis, Pantelis Sarafidis

**Affiliations:** 1 Nephrology, Hippokration Hospital, Aristotle University of Thessaloniki, Thessaloniki, GRC; 2 Physical Education and Sports Science, Aristotle University of Thessaloniki, Serres, GRC

**Keywords:** autonomic function, baroreflex sensitivity, hd (hemodialysis), hemodynamic parameters, peritoneal dialysis (pd)

## Abstract

Introduction

Cardiac arrhythmias are the leading mortal cause of end-stage kidney disease (ESKD), and autonomic dysfunction plays a predominant role. This is the first study to compare baroreflex sensitivity (BRS) and hemodynamic responses at rest and after mental and physical stimulation maneuvers between hemodialysis (HD) and peritoneal dialysis (PD) patients.

Methods

A total of 68 ESKD patients (34 HD and 34 PD, matched for age, sex, and dialysis vintage) were included. Continuous recordings from Finometer-PRO at rest and during mental arithmetic, orthostatic, and handgrip exercise tests were used for the calculation of BRS and hemodynamic responses in each individual.

Results

The two groups were similar in terms of age, sex, dialysis vintage, and major comorbidities. BRS during mental (HD: 3.59±2.62 vs. PD: 5.50±9.40 ms/mmHg, p=0.280) and physical stress tests (orthostatic: HD: 3.23±2.42 vs. PD: 2.07±2.69 ms/mmHg, p=0.777) was similar between HD and PD patients. During the mental test, both groups presented increases in systolic (SBP) and diastolic blood pressure (DBP) levels compared to rest (SBP HD: 156.3±27.7 vs. 142.7±20.0 mmHg, p<0.05; PD: 158.0±25.6 vs. 143.1±23.6 mmHg, p<0.05), but without significant between-group differences (p=0.853/p=0.611). Similarly, no significant between-group differences were noted in the other hemodynamic parameters. Mean SBP levels during the orthostatic test were significantly decreased compared to rest in both groups (HD: 135.4±26.8 vs. 142.2 ±20.1 mmHg; p<0.05; PD 135.3±21.9 vs. 143.1±23.6 mmHg; p<0.05), but the overall response was not different between groups (p=0.937). Finally, the hemodynamic responses during handgrip exercise and recovery did not differ between HD and PD.

Conclusions

BRS and hemodynamic responses to mental and physical stress tests were similar between HD and PD patients, suggesting that the function of the autonomic nervous system (ANS) in ESKD is not affected by dialysis modality.

## Introduction

Cardiovascular events are the primary cause of death among patients with end-stage kidney disease (ESKD) [1], with cardiac arrhythmias and sudden cardiac arrest accounting for over 50% of these fatalities [1]. This remarkably elevated cardiovascular burden can be explained by the presence of traditional (i.e. older age, hypertension, diabetes, etc.) and non-traditional, specific for ESKD, risk factors [2]. Among the latter, autonomic nervous system (ANS) dysfunction is regarded as a key factor in the development of an arrhythmogenic substrate in patients with ESKD [2].

The assessment of ANS function is challenging [3]. As of this writing, multiple functional methods have been utilized to assess the integrity of ANS at rest and after stimulation tests [3]. Testing the autonomic cardiovascular reflexes triggered by specific provocative tests (e.g., standing/orthostatic test, mental tests, exercise, and Valsalva maneuver) have been widely used in the literature, as they allow to detect minimal alterations in ANS function, that are not evident at resting conditions [4].

In this context, baroreflex sensitivity (BRS) emerged as a valuable, non-invasive, and reliable tool for assessing the autonomic control of the cardiovascular system that has been shown to have strong associations with hard cardiovascular outcomes in different populations [5]. In particular, in patients after myocardial infarction, low BRS was associated with greater risk for cardiac arrests [6], while in patients with stroke, low BRS was linked to increased all-cause mortality [7]. Most importantly, in ESKD patients, reduced BRS and BEI are independent predictors of all-cause mortality and sudden death [8].

The differential impacts of the two dialysis modalities (i.e., hemodialysis (HD) and peritoneal dialysis (PD)) on ANS function remain insufficiently explored. It has been established that the HD session per se promotes abnormal ANS function [9-12]. As such, it has been postulated that PD patients may present more favorable ANS responses than those undergoing HD, as the intermittent nature of HD represents a less “physiological” kidney replacement modality compared to PD [13]; on the contrary, PD is a continuous modality without rapid volume and solute alterations during treatments. A few preliminary studies assessing the extent of ANS dysfunction in HD vs. PD patients showed similarly impaired ANS function as assessed through heart rate (HR) responses to simple autonomic tests [14], nerve conduction studies, sympathetic skin response (SSR) [15], and iodine-123 metaiodobenzylguanidine (^123^I-mIBG) myocardial scintigraphy [16]. In a recent study from our group, no differences in HR variability (HRV) indices at rest and after mental and physical stimulation were found between HD and PD patients, but the ANS responses following the sit-to-stand test were more impaired in HD [17].

To our knowledge, despite efforts to evaluate ANS dysfunction in ESKD, no study to date has compared BRS and hemodynamic parameters at rest and in response to physical and mental stress in HD and PD patients. Thus, given the known physiological differences between these modalities, this study aimed to directly compare BRS and hemodynamic responses at rest and after mental and physical stimulation maneuvers between HD and PD patients.

## Materials and methods

Study participants

This secondary analysis of a previous study [17] recruited 68 ESKD patients (34 on HD and 34 on PD, matched in a 1:1 ratio for age, sex, and dialysis vintage) from the First Department of Nephrology, Hippokration Hospital, Aristotle University of Thessaloniki, Greece, and affiliated units. The study protocol (NCT05278702) was reviewed and approved by the Ethics Committee of the School of Medicine, Aristotle University of Thessaloniki (approval number 2433). All procedures were performed according to the Declaration of Helsinki (2013 Amendment) and all participants provided informed written consent prior to study enrollment. The study followed a cross-sectional design.

Inclusion criteria were i) age >18 years; ii) ESKD treated with HD or PD for more than three months; iii) for HD patients, standard schedule of thrice-weekly dialysis sessions; and iv) provision of informed written consent. Exclusion criteria included i) changes in antihypertensive, cardiovascular, or neurological treatment one month prior to study enrollment; ii) history of hereditary or degenerative neurological disorders (e.g., Parkinson’s disease and multiple sclerosis) that cause primary ANS dysfunction; iii) history of ANS dysfunction due to acquired disorders (e.g., diabetes mellitus, amyloidosis, autoimmune diseases, and spinal cord injuries); iv) active malignancy or other comorbidity linked to poor prognosis; v) active infection or relevant intercurrent illness; and vi) history of alcohol or drug abuse or severe mental disorder. 

Study design and data collection

Eligible HD patients were evaluated in our research center on a non-dialysis day, with the measurements being performed either 24-h prior to or 24-h after the middle of a mid-week HD session in a random order (the randomization of the order was performed through software). PD patients were assessed on the day of their routine evaluation at the PD Unit. Prior to ANS function measurements, baseline demographics, anthropometrics, medical history, and concomitant medications were recorded, and a detailed physical examination was performed. Venous blood samples for routine laboratory tests were obtained, immediately before the HD session that preceded or followed the day of the measurements for the HD patients or immediately before the study assessments for the PD patients. All procedures took place in a room with an ambient temperature of 23-24°C.

**Figure 1:**
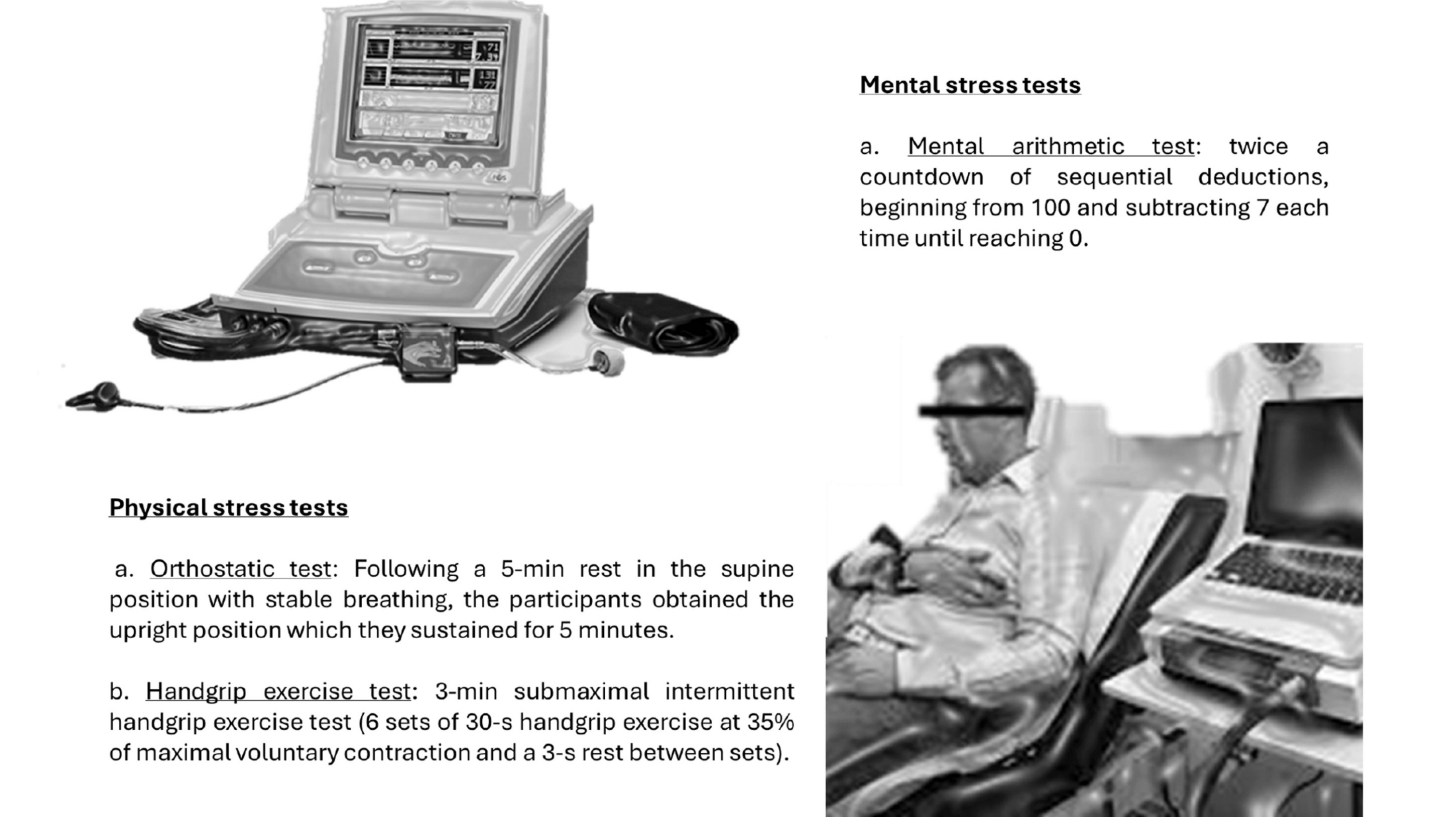
Assessment of ANS function parameters at rest and after stimulation with mental and physical stress tests with the use of the photoplethysmograph apparatus. ANS, autonomic nervous system This figure represents the original effort of the authors.

After explanation and familiarization with the study procedures, participants were connected to a photoplethysmograph apparatus (Finometer-PRO, Finapres Medical Systems, The Netherlands) (Figure 1). The apparatus was placed on the third finger of their non-dominant arm for continuous measurement of beat-to-beat blood pressure (BP) and HR. Then, the participants obtained the semi-reclined position and were advised to relax during the initial equipment calibration (about 12-15 min). After 5 min of rest (baseline measurement), the patients underwent the ANS stimulation tests presented in Figure 1, as described previously in detail [17].

Data analysis and ANS function parameters calculation

Hemodynamic parameters (i.e., mean arterial pressure (MAP), stroke volume (SV), cardiac output (CO), total peripheral resistance (TPR)) were calculated using the BeatScope software (Beatscope version 1a, Finometer, Finapres Medical Systems). BRS (ms/mmHg) was assessed by examining the spontaneous fluctuations in the BP as assessed by the cross-correlation method, using the same software [18].

Statistical analysis

Statistical analysis was performed with the Statistical Package for Social Sciences (SPSS Inc., Chicago, IL, USA) version 22.0. Data were analyzed by an investigator blinded to patient group (HD vs. PD) classification. Continuous variables are expressed as mean ± standard deviation (mean±SD) or median (interquartile range), depending on the normality of distribution, as assessed by the Shapiro-Wilk test. Categorical variables are presented as absolute frequencies and percentages (n, %). Between-group comparisons for continuous variables were performed with the independent Student’s t-test or the Mann-Whitney U test, where applicable. Categorical variables were compared using the Chi-square test. To evaluate the responses of BRS and hemodynamic parameters during the ANS tests, two-way analyses of variance (ANOVA) with repeated measures on the group (HD vs. PD) by evaluation time points (i.e., at rest, during the test and/or recovery) were implemented, followed by Tukey’s post hoc test for pairwise comparisons. For all comparisons, a p-value of <0.05 (two-tailed) was considered statistically significant.

## Results

Baseline characteristics

Table 1 presents the baseline characteristics of the 34 HD and 34 PD patients included in the analysis. No differences were observed between the two groups in terms of age, sex, and dialysis vintage; PD patients presented higher values of body mass index (BMI) than HD. Furthermore, no between-group differences in any major comorbidities were detected, with the exception of heart failure, being more common in HD patients. With regards to laboratories and concomitant medication, serum urea, potassium, and calcium levels were higher in HD patients, while the use of renin-angiotensin blockers was more common in PD patients (Table 1). 

**Table 1:** Baseline characteristics of HD and PD patients. Continuous variables are reported as mean±standard deviation (mean±SD) or median (interquartile range) based on the normality of distribution tested with the Shapiro-Wilk test. Categorical variables are presented as absolute frequencies and percentages (n, %). Between-group comparisons for categorical variables (i.e., male gender, major comorbidities, antihypertensive agents, and erythropoietin-stimulating agents) are made using the Chi-square test and for non-normally distributed variables (i.e., dialysis vintage and parathormone) with Mann-Whitney U test. The rest comparisons for normally distributed variables are performed by applying the independent Student’s t-test. For all comparisons, a value of p<0.05 is deemed as statistically significant. ACEi, angiotensin converting enzyme inhibitor; ARBs, angiotensin 2 receptor blocker; BMI, body mass index; CAD, coronary artery disease; CCB, calcium channel blockers; HD, hemodialysis; PD, peritoneal dialysis

Parameter	HD	PD	P-value
N	34	34	
Age (years)	57.0±15.5	57.0±15.7	0.997
Males (n, %)	18 (52.9%)	18 (52.9%)	1.000
Dialysis vintage (months)	73.5 (77.9)	49.7 (44.6)	0.102
BMI (kg/m^2^)	25.0±3.7	27.3±4.9	0.035
Major comorbidities			
Diabetes (n, %)	3 (8.8%)	6 (17.6%)	0.476
CAD (n, %)	5 (14.7%)	6 (17.6%)	0.742
Heart failure (n, %)	12 (35.3%)	5 (14.7%)	0.050
Hypertension (n, %)	30 (88.2%)	33 (97.1%)	0.163
Dyslipidemia (n, %)	13 (38.2%)	18 (52.9%)	0.223
Antihypertensive treatment			
Number of antihypertensives	1.44±1.21	1.97±1.24	0.080
ACEi/ARB (n, %)	10 (29.4%)	21 (61.8%)	0.007
CCB (n, %)	16 (47.1%)	13 (38.2%)	0.462
Beta blocker (n, %)	19 (55.9%)	25 (73.5%)	0.128
Alpha blocker	3 (8.8%)	0 (0.0%)	0.239
Diuretic (n, %)	11 (32.4%)	19 (55.9%)	0.051
Central acting (n, %)	0 (0.0%)	3 (8.8%)	0.239
Erythropoietin-stimulating agents (n, %)	27 (79.4%)	20 (58.8%)	0.06
Laboratory values			
Hemoglobin (g/dL)	11.27±1.17	11.42±1.68	0.689
Urea (mg/dL)	136.8±38.3	112.9±22.2	0.003
Creatinine (mg/dL)	8.95±2.55	8.20±2.90	0.262
Sodium (mmol/L)	139.4±2.4	138.6±3.3	0.274
Potassium (mmol/L)	5.16±0.63	4.54±0.63	<0.001
Calcium (mg/dL)	9.26±0.70	8.84±0.55	0.008
Phosphate (mg/dL)	4.51±0.88	4.77±0.94	0.252
Parathormone (ng/L)	268.3 (260.1)	307.6 (193.8)	0.619

BRS and hemodynamic parameters at rest

The comparison of BRS and hemodynamic parameters at rest between HD and PD patients is presented in Table 2. As shown in Table 2, BRS did not differ between HD and PD patients in resting conditions (4.41±3.16 vs. 5.89±8.21 ms/mmHg, p=0.330). Furthermore, no differences in SBP, DBP, and MAP levels were observed; resting HR was higher in HD patients compared to PD patients (73.9±10.9 vs. 69.0±7.6 bpm, p=0.035). No significant differences in the resting hemodynamic parameters (SV, CO, and TPR) were detected (Table 2). 

**Table 2:** BRS and hemodynamic parameters at rest in HD and PD patients. All continuous variables are normally distributed (tested with the Shapiro-Wilk test) and reported as mean±standard deviation (mean±SD). Between-group comparisons are made using the independent Student’s t-test. For all comparisons, a value of p<0.05 is deemed as statistically significant. BRS, baroreflex sensitivity; CO, cardiac output; DBP, diastolic blood pressure; HD, hemodialysis; HR, heart rate; MAP, mean arterial pressure; PD, peritoneal dialysis; SBP, systolic blood pressure; SV, stroke volume; TPR, total peripheral resistance

Parameter	HD	PD	P-value
SBP (mmHg)	142.7±20.0	143.1±23.6	0.940
DBP (mmHg)	72.7±9.2	74.3±10.1	0.497
MAP (mmHg)	100.0±12.3	101.2±14.2	0.711
HR (beats/min)	73.9±10.9	69.0±7.6	0.035
SV (mL)	104.3±29.1	107.9±35.5	0.649
CO (L/min)	7.6±2.2	7.4±2.3	0.715
TPR (mmHg.min/L)	0.87±0.28	0.92±0.33	0.503
BRS (ms/mmHg)	4.41±3.16	5.89±8.21	0.330

BRS and hemodynamic parameters during the mental stress test

**Figure 2:**
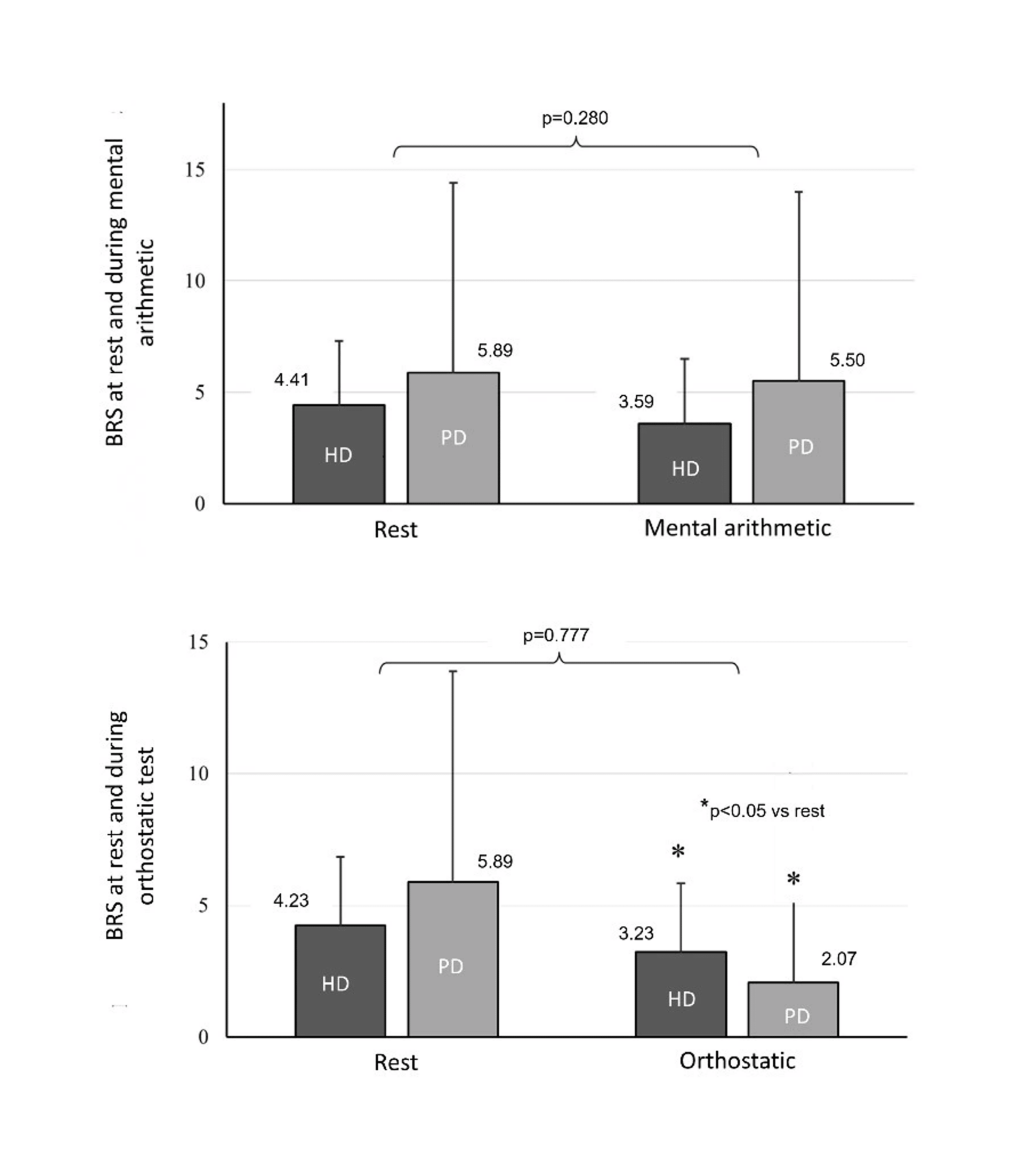
BRS at rest and during the mental arithmetic and orthostatic tests. Responses of BRS during the mental and orthostatic tests are evaluated with two-way ANOVAs with repeated measures on the group (HD vs. PD) by evaluation time points (i.e., at rest and during the test and/or recovery). For pairwise comparisons, Tukey’s post hoc tests are applied. For all comparisons, a value of p<0.05 is deemed as statistically significant. ANOVA, analysis of variance; BRS, baroreflex sensitivity; HD, hemodialysis; PD, peritoneal dialysis

**Figure 3:**
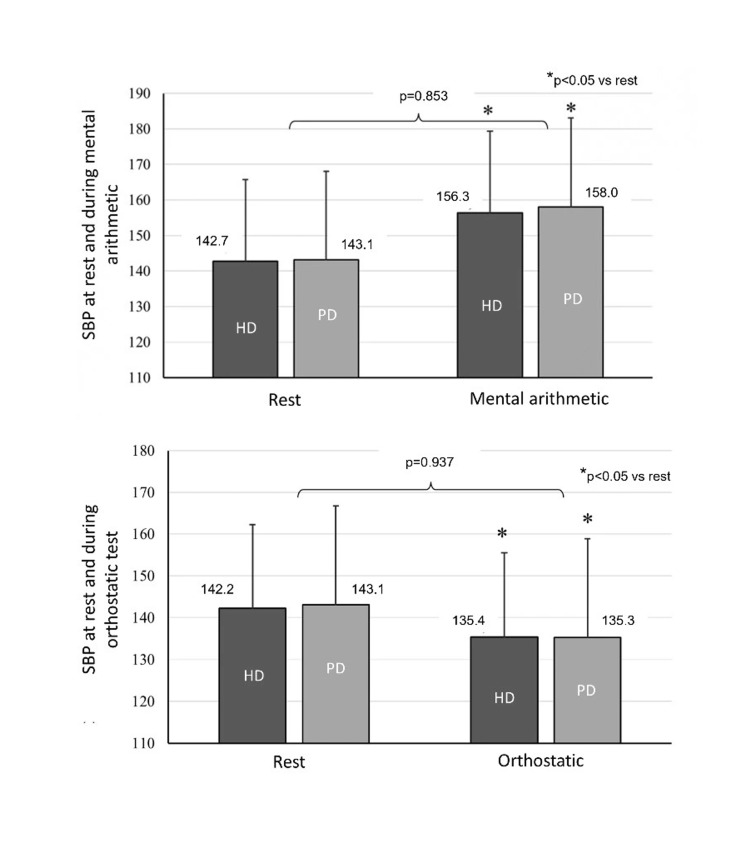
SBP at rest and during the mental arithmetic and orthostatic tests. Responses of SBP during the mental and orthostatic tests are evaluated with two-way ANOVAs with repeated measures on the group (HD vs. PD) by evaluation time points (i.e., at rest and during the test and/or recovery). For pairwise comparisons, Tukey’s post hoc tests are applied. For all comparisons, a value of p<0.05 is deemed as statistically significant. ANOVA, analysis of variance; HD, hemodialysis; PD, peritoneal dialysis; SBP, systolic blood pressure

During the mental arithmetic test, BRS did not show significant changes compared to rest in both groups (HD during mental test vs. resting, 3.59±2.62 vs. 4.41±3.16 ms/mmHg, and PD: 5.50±9.40 vs. 5.89±8.21 ms/mmHg, p>0.05 for both); no between-group differences were detected (p=0.280) (Figure 2). With regards to hemodynamic responses, SBP and DBP levels were increased in both HD and PD patients compared to rest (SBP HD: 156.3±27.7 vs. 142.7±20.0 mmHg, p<0.05; PD: 158.0±25.6 vs. 143.1±23.6 mmHg, p<0.05, respectively), but without significant between-group differences (p=0.853 and p=0.611 for SBP and DBP, respectively) (Figure 3). Similar were the results for MAP (HD: 109.6±19.4 vs. 100.0±12.3 mmHg, p=0.018; PD: 111.7±16.4 vs. 101.2±14.2 mmHg, p=0.006; between-group comparison p=0.644). HR was significantly increased during the mental test (HD: 80.4±13.1 vs. 73.9±10.9 bpm; p<0.05, PD: 73.9±9.5 vs. 69.0±7.6 bpm; p<0.05), with PD patients presenting statistically lower HR values (p=0.023). Regarding SV, CO, and TPR, no significant within- (from baseline) and between-group differences were noted during this testing period. 

Hemodynamic parameters and BRS during physical stress tests

Orthostatic Test

During the orthostatic test, BRS was significantly reduced compared to baseline in both groups (HD: 3.23±2.42 vs. 4.23±3.03 ms/mmHg, p<0.05; PD: 2.07±2.69 vs. 5.89±8.21 ms/mmHg, p<0,05); however, the overall response did not differ between HD and PD patients (p=0.777) (Figure 2). Similarly, the mean SBP (HD: 135.4±26.8 vs. 142.2±20.1 mmHg; p<0.05; PD: 135.3±21.9 vs. 143.1±23.6 mmHg; p<0.05) (Figure 3) and MAP levels during the orthostatic test were significantly decreased from baseline, but the overall response was not different between groups (p=0.937). HR was significantly increased during the orthostatic test in both groups (HD: 84.5±13.1 vs. 73.8±11.1 bpm; p<0.05; PD: 79.9±10.0 vs. 69.0±7.6 bpm p<0.05) with similar values between HD and PD patients (p=0.061). DBP, SV, CO, and TPR were not significantly different from baseline and the corresponding changes were similar between HD and PD. 

Handgrip Test

During the handgrip test, BRS was statistically lower compared to baseline in both HD and PD patients (HD: 4.10±2.64 vs. 3.23±1.97 ms/mmHg p<0.05; PD: 5.99±8.32 vs. 3.96±4.31 ms/mmHg, p<0.05), without significant changes in BRS at the 3-min recovery period compared to during the handgrip test. When comparing BRS levels between the two groups, no significant differences were detected during the handgrip test and the recovery period (p=0.221). Mean SBP, DBP, and MAP levels were statistically higher compared to baseline during the handgrip test; following the recovery period, they all decreased compared to values during the test. SV and TPR were significantly altered from baseline during the handgrip test and the recovery period in both groups (SV: HD: 103.0±28.6 vs. 102.1±38.1 vs. 98.6±33.0 mL, p<0.05, PD: 107.9±35.5 vs. 95.3±38.6 vs. 96.2±38.4 mL, p<0.05; TPR: HD: 0.88±0.28 vs. 1.0±0.45 vs. 0.93±0.42 mmHg.min/L, p<0.05, PD: 0.92±0.33 vs. 1.14±0.49 vs. 1.08±0.48 mmHg.min/L, p<0.05, resting vs. handgrip test vs. recovery, respectively). The responses of all hemodynamic parameters during the handgrip test and the recovery period were similar between HD and PD patients.

## Discussion

This is the first study comparing BRS and hemodynamic parameters in HD and PD patients both at rest and in response to mental and physical stress tests. BRS did not change from baseline during the mental stress test but was statistically lower in both groups during the physical stress tests; mean BRS values in HD vs. PD patients were similar during all ANS tests. Regarding the hemodynamic parameters, during the mental test, both groups had higher SBP/DBP levels compared to the rest, without demonstrating significant between-group differences. Similarly, no significant between-group differences were evident in SV, CO, or TPR during the mental test. Moreover, mean SBP levels during the orthostatic test were significantly decreased compared to rest in both groups, but the overall response was not different between groups. Finally, during handgrip exercise and recovery, the hemodynamic responses were not different between HD and PD patients.

ANS dysfunction is a common feature of chronic kidney disease, with its prevalence and severity gradually increasing with the disease progression toward ESKD [19,20]. In this population, ANS dysfunction is generated by the synergistic effects of multiple mechanisms [3]. Uremic toxins exert direct actions through modulating neurohumoral regulatory nuclei and reinforcing afferent stimuli, which prompt sympathetic overactivity and baroreflex dysfunction [21,22]. In parallel, ESKD-related fluid and electrolyte imbalances damage nerve fibers through swift alterations in the endoneurial space, disrupt action potentials, and impair neurotransmission [23]. These alterations are even more pronounced in HD patients, as the intermittent nature of HD therapy per se has been associated with marked damage in nerve fibers and the baroreceptors [23]. Furthermore, abnormal vascular remodeling and arterial stiffness impair baroreceptor control [24], while cardiac remodeling and hypertrophy blunt volume-sensitive and chemosensitive cardiopulmonary reflexes by desensitizing the corresponding receptors [24,25]. Other contributing mechanisms include overactivation of the renin-angiotensin-aldosterone system, increased oxidative stress and inflammation, endothelial dysfunction, metabolic acidosis, anemia, and insulin resistance [3].

Studies comparing ANS function between different dialysis modalities are limited, and most involve small sample sizes. In a preliminary analysis including 12 HD patients, 10 PD patients, and 12 healthy controls, both HD and PD patients exhibited blunted HR responses following the Valsalva maneuver, deep breathing, and standing tests compared to controls, but no differences were reported between HD and PD groups [14]. In another study using resting gated myocardial perfusion and ¹²³I-mIBG myocardial scintigraphy, indices of global cardiac sympathetic activity (early and delayed heart-to-mediastinum ratios (eHMR and dHMR, respectively) and myocardial washout rate (WR)) were similar between HD and PD participants [16]. Moreover, in a study employing nerve conduction techniques and SSR in 20 HD and 24 PD patients, sensory and motor nerve conduction velocities were comparable in both dialysis groups; however, a higher percentage of HD patients showed impaired SSR, suggesting a potentially higher prevalence of subclinical neuropathy in this group [15]. In a recent study from our group involving 68 ESKD patients (34 HD and 34 PD), linear and non-linear HRV indices, both at rest and in response to mental and physical stimulation (orthostatic, sit-to-stand, and handgrip exercise tests), did not differ significantly between HD and PD, except for HRV indices following the sit-to-stand test, which were more impaired in the HD group [17]. Finally, in the largest study to date comparing baroreflex function in 95 HD and 59 PD patients, both BRS and BEI were similar between the two groups but significantly impaired compared to age-matched healthy subjects [26].

To the best of our knowledge, this is the first study to compare BRS and hemodynamic parameters between HD and PD patients, both at rest and in response to physical and mental stress tests. We hypothesized that PD patients would likely exhibit less impaired ANS function, given that the intermittent nature of HD sessions is a significant factor contributing to ANS imbalance [12]. A key strength of this study is our decision to evaluate ANS function not only at rest but also under multiple stimulation tests. This approach allows us to detect subtle ANS dysregulations that might otherwise go unnoticed during resting conditions [12]. Our findings are generally consistent with previous studies in this field; we observed no significant differences in BRS and hemodynamic responses between HD and PD patients, both at rest and during stress tests. This similarity could be partially attributed to the multifactorial nature of ANS dysfunction in ESKD, where the underlying mechanisms may be comparably disrupted across both dialysis modalities, leading to similar ANS responses. As we have previously reported, many of the contributing factors, including volume overload, vascular function, total small solute clearance, BP control and variability, subclinical inflammation, anemia prevalence, and cardiac structure and function, are largely similar between the two dialysis modalities [13,27-29]. 

There are some limitations that should be acknowledged. First, our study included a relatively small number of participants; but this is a common challenge in studies in this field and our study is one of the largest up to date. Additionally, patients receiving β-blockers were not excluded from the study; β-blockers are known for modifying ANS function and consequently, their use may have affected the results of the performed ANS stimulatory maneuvers. Given that more than 50% of ESKD patients receive β-blockers for various comorbidities (e.g., hypertension, coronary artery disease, heart failure, and atrial fibrillation) [30], designing a study with β-blocker-naïve patients would not accurately represent the broader ESKD population.

## Conclusions

In conclusion, this study demonstrated that there were no significant differences between HD and PD patients in BRS and hemodynamic parameters, neither at rest nor after stimulation through mental and physical stress tests. These results further expand existing evidence suggesting that ANS function in ESKD is not affected by the type of dialysis modality and that the severity of ANS dysfunction is comparable between HD and PD patients. However, given that existing modest differences might have not been detected, larger studies performing additional ANS function maneuvers should confirm our findings. In the near future, studies examining possible associations of ANS dysfunction with important clinical parameters and cardiovascular outcomes in ESKD patients could prove useful in stratifying and overall managing these high-risk patients.
